# Pediatric thyroid surgery: Retrospective analysis on the first 25 pediatric thyroidectomies performed in a reference center for adult thyroid diseases

**DOI:** 10.3389/fendo.2023.1126436

**Published:** 2023-03-03

**Authors:** Francesco Quaglino, Alex Bruno Bellocchia, Gerdi Tuli, Jessica Munarin, Patrizia Matarazzo, Luca Cestino, Federico Festa, Giulia Carbonaro, Salvatore Oleandri, Claudia Manini, Riccardo Vergano, Luisa De Sanctis

**Affiliations:** ^1^ General Surgery Division, Maria Vittoria Hospital, Turin, Italy; ^2^ Pediatric Endocrinology Division, Regina Margherita Children’s Hospital, Turin, Italy; ^3^ Endocrinology Division, Maria Vittoria Hospital, Turin, Italy; ^4^ Pathology Division, Maria Vittoria Hospital, Turin, Italy; ^5^ Otorhinolaryngology Division, Maria Vittoria Hospital, Turin, Italy

**Keywords:** thyroidectomy, children, complications, transient hypoparathyroidism, IONM

## Abstract

**Introduction:**

Pediatric thyroid carcinoma represents about 4-5% of all pediatric carcinoma with an incidence of 0.5 cases/100,000, compared to 2-10/100000 cases in the adult population. The aim of this study is to present the experience of a reference adult endocrine surgery unit in charge of the treatment of pediatric thyroid diseases.

**Materials and methods:**

From January 2019 to September 2022, 25 patients, aged 5-17, underwent thyroid surgery. We analysed indications for surgery, use of intraoperative nerve monitoring (IONM), definitive histological examination, postoperative outcomes and risk factors related.

**Results:**

Surgical indication was performed for Graves’ disease (27%) and for nodular pathology (73%): of these, four were malignant lesions (TIR4/TIR5), eight with indeterminate characteristics (TIR3A/TIR3B) and four characterized as benign (TIR1/TIR2). Total thyroidectomy (TT) was performed in 76% of cases, three of which were prophylactic for the activation of the RET gene mutation in MEN 2A. IONM was used in eight cases (32%), all patients aged 11 years or less. FNA’s accuracy was 100% for lesions typified as benign and malignant (TIR1/TIR2 and TIR4/TIR5). The overall malignancy rate achieved was 40% and in the final histological examination 75% of the TIR 3B lesions were malignant. Six patients (24%) developed hypoparathyroidism in the first postoperative day, with normalization of calcium values within thirty days in 5 patients.

**Conclusions:**

Pediatric thyroid nodules are rare and distinguished from adult thyroid disease by a worse prognosis and higher malignancy rates. Our work reports a much higher malignancy rate among indeterminate TIR 3B lesions than observed in the adult population and the three patients who underwent prophylactic total thyroidectomy for activating RET gene mutation had all a definitive histological diagnosis of medullary carcinoma. Post-surgical hypoparathyroidism is a common finding in these patients: in most cases the condition is transient and it benefits from supportive therapy. Intraoperative finding of a thinner recurrent laryngeal nerve in younger patients makes nerve isolation more difficult than in adult surgery: IONM is recommended in patients under 12. Pediatric thyroid surgery is challenging, we sustain it requires referral thyroid Centers for thyroid disease with highly skilled general endocrine surgeons.

## Introduction

Pediatric thyroid diseases of surgical interest are a rare entity and are represented by both benign and malignant conditions (1). The incidence of thyroid nodules and thyroid cancer in children not only appears to be steadily increasing but also demonstrates a worse prognosis. According to some studies recently published, the risk of malignancy of pediatric thyroid nodules varies from 9 to 50% with an average risk of 26% (2–4), far higher than the expected value in the adult population (5%) (2–5).

Transient hypoparathyroidism and inferior laryngeal nerve injury (recurrent laryngeal nerve) are the most described conditions in literature: however, incidence data still remain limited due to the absence of randomized control studies and several meta-analyses (6–8).

In clinical practice, the management of pediatric thyroid gland largely follows the adult thyroid guidelines. Only the 2015 guidelines of the American Thyroid Association (ATA) have provided a first initial contribution, defining protocols and providing indications for the management of thyroid nodules in children and particularly for the use of intraoperative nerve monitoring (IONM) (9, 10).

This study aims to report the experience of the Reference Center for Thyroid Neoplasms and Endocrine Glands of Regional Oncology Network of the Maria Vittoria Hospital in Turin in the treatment of pediatric thyroid disease: is the pediatric thyroid the exclusive prerogative of the pediatric surgeon? Starting from our results as a Center of Reference for adult endocrine surgery and reviewing data in the literature, according to which the discriminating factor remains surgical competence, we have tried to answer the initial question.

We conducted a retrospective study analyzing a population of children undergoing thyroid surgery to investigate indications for surgery, type of surgical interventions, IONM use and patient outcomes. We also examined risk factors recognized in literature for the development of transient postoperative hypoparathyroidism comparing their relevance in adult and pediatric population.

## Materials and methods

The work stems from the collaboration between the endocrine surgical unit of Maria Vittoria Hospital of Turin and the Regina Margherita Pediatric Hospital of Turin: a retrospective review was conducted on all the data collected over a period of 44 months relating to pediatric patients undergoing thyroid surgery.

Data were collected on patients under the age of 17 undergoing thyroid surgery at our Center according to the indications provided by the Pediatric Endocrinologists. Patients inclusion criteria were the age below 18 years and the presence of benign or malignant thyroid disease with surgical indication. Previous thyroid surgery was not an exclusion criterion from the study; patients with prior parathyroid disease or prior calcium homeostasis disorders were excluded to avoid subsequent selection confounding factors.

All the pediatric patients were treated by the same surgical team (two surgeons of which the first operator with an average of 80 thyroids/year and the second with an average of 40). Surgical techniques included the total thyroidectomy (TT), the thyroid lobectomy (LT) and the completion thyroidectomy with dissection of the central cervical compartment if intraoperative finding of adenopathies.

A unique selected pathologist’s team have managed the cytological and histopathological diagnosis. Patient demographics, type of intervention, IONM use and results obtained were collected and divided into databases.

Postoperative complications and potential risk factors for the development of transient hypoparathyroidism were examined. To define transient hypoparathyroidism, we used the definition of the European Society of Endocrinology Clinical Guideline (11), that describes a condition characterized by biochemical hypocalcemia (ionized Ca 2 + <1.16 mmol/l) and inappropriate PTH levels for at least 4 weeks (12, 13).

Hypocalcemia occurring after surgery was treated with calcium and vitamin D. The remission of transient hypoparathyroidism was defined with normalization of ionized Ca2+ in at least two consecutive measurements and in absence of integrative therapy.

All patients had at least a three months-follow-up with five post operative controls with laboratory tests and clinical screening. Measurements of ionized calcium and PTH levels were performed in the same time schedule. Calcium and PTH values were measured systematically the day before surgery (to rule out a previous disorder of calcium homeostasis), the day after surgery, at discharge, one month after discharge and three months later. All patients were evaluated by adult’s endocrinologist before hospital discharge, and after seven days by pediatric endocrinologist. Patients who needed supportive therapy underwent endocrinological screening one month after discharge that confirmed or not the normalization of serum calcium levels.

Statistical analyzes were performed with MedCalc software: for the dichotomous variables, Fisher’s test was applied. By convention, the significance threshold of P value was set at 0.05.

## Results

Over a period of 44 months, from January 2019 to September 2022, a total of 25 patients were enrolled for the study group: ten males (40%) and fifteen females (60%). Children were aged between 5 and 17 years, (mean age of 12.64 ± 3.86 SD) with almost 50% of cases in age range between 9-14 years (12 cases).

Total thyroidectomy (TT) was performed in 20 patients (80%), thyroid lobectomy (LT) in 5 patients (20%). In only one case, our team performed a radicalization with completion thyroidectomy, after a previous partial thyroidectomy that patient had undergone at another hospital Center. In three malignant cases we performed a total thyroidectomy with simultaneous unilateral lymph node dissection of the central cervical compartment (CND) for intraoperative detection of suspected unilateral cervical adenopathies.

The surgical indication was in 73% of cases (n=16) for thyroid nodules, in 27% (n=6) for Graves’ disease. In three cases we performed a total thyroidectomy for MEN 2A syndrome (**Table 1**).

**Table 1:** Demographics.

Patients	N (%)
Age (mean, SD)	12.64 ± 3.86
Male	10 (40)
Female	15 (60)
**Total thyroidectomy**	**20** (80)
Predisposing condition: MEN 2A Medullary Thyroid Carcinoma	3 (12)
Graves’ disease	6 (24)
Multinodular thyroid goiter	4 (16)
Papillary thyroid cancer (PTC)	7 (28)
Follicular thyroid cancer (FTC)	()
**Lobectomy**	**5** (20)
Uninodular thyroid goiter	3 (12)
Papillary thyroid cancer (PTC)	1 (4)
Completion thyroidectomy (in previous PTC)	1 (4)
**CND**	4 (16)
Unilateral	4 (16)
Bilateral-	
**Total**	**25** (100%)

Age reported in years. SD, standard deviation; MEN, multiple endocrine neoplasia; PTC, papillary thyroid cancer; FTC, follicular thyroid cancer.

All the patients underwent preoperative ultrasound to evaluate thyroid and neck. The preoperative procedure also included scintigraphy and fine needle aspiration when necessary based on the characteristics of the thyroid nodule. Preoperative laryngoscopy was scheduled to complete the diagnostic process.

Fine needle aspiration (FNA) was performed on the basis of nodule size and ultrasound features. A total of 16 patients (64%) underwent FNA with the results shown in **Table 2**. FNA showed an overall accuracy level of 100% for benign and malignant lesions (TIR 1/TIR 2 and TIR 4/TIR 5). Indeterminate TIR 3A lesions were found to be benign on definitive histology (goiter); in contrast, 75% of TIR 3B lesions were found to be malignant. Only in one case, the TIR 3B lesion was histologically benign, with a probable follicular adenoma on thyroid goiter. Three patients with RET mutation underwent prophylactic total thyroidectomy with the histological diagnosis of medullary thyroid cancer (all three patients were 6 years old or less); the preoperative calcitonin levels were respectively 3.4 pg/ml, 7 pg/ml and 2.2 pg/ml (mean 4.2 pg/ml).

**Table 2:** Preoperative diagnostics.

Preoperative diagnostics	N (%)
FNA	16 (64) … benign malignant
TIR 1	2 (12.5) … 2 0
TIR 2	2 (12.5) … 2 0
TIR 3A	4 (25) … 4 0
TIR 3B	4 (25) … 1 3
TIR 4	2 (12.5) 0 2
TIR 5	2 (12.5) 0 2
Accuracy FNA	15 (94)
Discordant FNA	1 (6)
No FNA:	9 (36)
Graves’ disease	6 (67)
MEN 2A Syndrome	3 (33)

FNA, fine needle aspiration; MEN 2A, multiple endocrine neoplasia. FNA classes: TIR 1c, non diagnostic; cystic type; TIR 2, benign; TIR 3A, low risk follicular damage; TIR 3B, follicular proliferation or suspected follicular neoplasia; TIR 4, suspected malignancy; TIR 5, malignancy.

The use of the IONM device was not systematic. The neuromonitoring was applied in 32% of cases and in eight surgical procedures (both total thyroidectomies and lobectomies). All patients were 11 years or younger and with documented disease malignant or suspected thyroid carcinoma. Intraoperative monitoring of the laryngeal nerve was performed using a specific pediatric, non-invasive and non-traumatic endotracheal tube.

The tube was coated by surfaced electrodes, which were placed between the vocal cords during intubation under visual control. Additional subdermal electrodes were placed over the shoulders. All electrodes were connected to the neuromonitoring device and the recurrent laryngeal nerve was identified with visual and sound feedback and stimulated before and after thyroid or parathyroid dissection.

The mean operative time between all the operations performed was 99 min, (total range 50- 143 min), with a difference of approximately 58 min between the operative times of a lobectomy and a total thyroidectomy (TT) performed for malignancy. The mean time to a thyroidectomy without IONM was 110 minutes compared to the same surgery with IONM of 122 minutes. A TT for benign pathology was on average 92 min, while malignant disease required an additional average of 32 min attributed to the addition of CND and greater surgical complexity. A TT for Graves’ disease was on average 114 min (**Table 3**).

**Table 3:** Operative time.

Procedure	Average operating time in min (range)
TT for malignancy	124 (80-146)
* With IONM	122 (115-143)
* Without IONM	110 (80-126)
TT for benign disease	92 (80-126)
TT for Graves’ disease	114 (90-126)
Lobectomy	66 (50-80)
* With IONM	72 (65-80)
* Without IONM	58 (50-66)

TT, total thyroidectomy; IONM, intraoperative nerve monitoring.

Results for postoperative complications are shown in **Table 4**. The mean hospital stay was 3.4 days (with a range of 2.6 to 4.5 days). None of the patients required a second surgical look to evacuate bleedings or hematomas, no recurrent nerve injury or paralysis was documented, either in the group of children undergoing IONM thyroidectomy or without.

**Table 4:** Postoperative complications.

Postoperative complications	N (%)
Bleeding or hematoma-	
Recurrent laryngeal nerve injury-	
**Hypocalcemia:**	**6 (24)**
** Transient hypoparathyroidism:**	5 (83) *
In: Multinodular thyroid goiter	2
Graves’ disease	1
Papillary thyroid carcinoma	1
Cowden Syndrome	1
** Persistent hypoparathyroidism:**	1 (17) **
In: Papillary thyroid carcinoma	1

*normalization of calcium levels within 30 days. **persistence of hypocalcemia in the third post operative month.

The incidence of post operative hypoparathyroidism was 24% (n=6). Six patients developed hypocalcemia the first day after surgery, with serum calcium values below the expected standard. Patients had extremely heterogeneous underlying thyroid disease: two multinodular thyroid goiters, one Graves’ disease, one Cowden’s syndrome and two papillary thyroid cancers. None of these patients had symptoms related to hypocalcemia (such as paresthesia, tetany, or convulsions), the diagnosis was only biochemical with mean serum calcium values of 8.2 mg/dl. All these patients started supplemental calcium and vitamin D therapy on the first postoperative day, and they were discharged on average on the third postoperative day like the other children. The finding of hypocalcemia did not lengthen hospital stay of patients who, unlike the others, were discharged with prescriptions.

Supportive therapy with oral calcium and calcitriol was continued for one month after discharge when it was stopped in cases of normalization of calcium levels. At the 3 months-check-up only one patient presented chronic hypoparathyroidism; on the contrary, in the remaining five cases (83%) serum calcium levels were regular on laboratory tests, even in absence of therapy.

Many risk factors for the development of postoperative hypoparathyroidism in adults are reported in literature. However, there is no accordance on the various parameters cited in different published studies, including young age (less than 12 years old), female sex, type of surgery performed (TT or lobectomy), underlying thyroid disease and the contextual or not CND. Likewise, it remains a matter of debate whether these parameters can also influence post-operative outcomes in a pediatric population.

In our work, a sufficiently high level of significance was not reached to be able to correlate the single factors with the subsequent development of transient hypoparathyroidism in the postoperative period. Therefore, there is no causal link between individual variables and post-operative outcome (**Table 5**).

**Table 5:** Risk factors for the development of postoperative hypoparathyroidism.

Variable	*P*-value
Female gender	0.59
Age under 12 years old	1
Thyroid disease *	0.51
Type of surgery * *	0.10
CND or not	0.29
Benign or malignant lesion	0.59

*Nodular lesion or not. **Total thyroidectomy or lobectomy.

## Discussion

Pediatric thyroid diseases of surgical interest are rare: however, data provided by literature report constantly increasing incidence rates (14, 15). Children and adolescents show diseases with a worse prognosis and higher malignancy rates (12.5-50%), much higher than the adult population by 5-10% (16).

The case studies we presented in our Center seem to confirm this result. First, our work reports a much higher malignancy rate among indeterminate TIR 3B lesions than observed in the adult population (75% versus 15-30% expected in adults). Similarly, the three patients who underwent prophylactic total thyroidectomy for activating RET gene mutation had a definitive histological diagnosis of medullary carcinoma.

In consideration of the worst prognosis of these pathologies, it is therefore necessary to have dedicated guidelines on the subject. If up to now pediatric thyroid pathology followed the same guidelines as adults, it is however necessary to have specific algorithms and operative protocols also for thyroid nodules in children and adolescents. The American Thyroid Association (ATA) guidelines were the first to be published in 2015 to define the management of pediatric thyroid nodules (9).

As reported by previous studies, surgical treatment of thyroid pathology requires not only multidisciplinary management (2) but also a close and “collaborative” skills both from endocrinologists and pediatric surgeons (17). However, it is still debated in literature which surgeon can best approach pediatric thyroids (1).. In fact, pediatric surgeons rarely perform thyroid surgery and, at the same time, endocrine surgeons rarely operate pediatric patients. In 2008, Tuggle and colleagues published their study in *Surgery* asking *“Should it be the endocrine surgery specialist who, in our experience, cares for an average of 3 children per year? Or should it be pediatric surgery that only performs 3 thyroid surgeries per year, an average? “* (18).

Likewise, Allison K et al. in 2021 reported in *Cancers* that pediatric thyroidectomy could be performed by pediatric and adult general surgeons as well as pediatric and adult otolaryngologists. Data published in the study show how in the period 1999-2017 pediatric thyroidectomies performed by general surgeons decreased from 60% to 18%, compared to an almost tripled increase in the same period as those performed by the pediatric surgeons (18-42%) (19). The same authors define in their study the concept of “surgeon volume” as the minimum acceptable number of thyroidectomies/year per operator and they demonstrate how this factor could positively impact on post operative outcomes. Therefore, high-volume surgeons are defined as those professionals with surgical cases exceeding thirty thyroidectomies per year (19). The “surgeon’s volume” seems to be the only factor associated with a better impact on patient outcomes: the literature reports the importance of the surgeon’s high operating volume and how this factor can contribute to the decrease in morbidity in high-risk surgical procedures (20). The demonstration of how the high-volume surgical Center, regardless of the surgical subspecialty involved, can offer to children, as in adults, the best results in terms of outcomes and complications is an evidence demonstrated by several studies (18–20).

This work fits into this context, in which the experience of general endocrine surgeon has been “tested” to surgically treat thyroid disease in children.

Over a total period of 44 months, from January 2019 to September 2022, 25 pediatric patients underwent thyroid surgery at our Center. The sample represented in our study, although in a limited number, remains in agreement with previous publications that appeared in the literature, with higher series of 75-200 patients, but collected in a period between 8 and 35 years (with an average of 6-9 children treated per year) (21–24).

Our sample of 25 children with an average value of 6.25 patients/year is therefore comparable to other works with larger case series, also considering the more difficult time context in which the study was conducted. In fact, from January 2020 to March 2022 the surgical activity of our Center was heavily affected by the period of the Sars-Cov2 pandemic and on several occasions our Center was destined for positive Covid patients, with the possibility of performing surgical interventions in regime of emergency.

A multidisciplinary approach was adopted in the management of patients: only at the end of the diagnostic phase performed by pediatricians and pediatric endocrinologists at the Regina Margherita Hospital patients were send for surgical evaluation to our surgical division. According to 2015 ATA guidelines, a thyroid ultrasound was performed preoperatively in all children and adolescents with palpable nodules, clinical features of suspicion and asymmetry of the gland. Similarly, the indication for ultrasound-guided FNA was made only in selected cases: anamnestic risk factors or clouded areas of thyroid parenchyma of unclear interpretation, or in presence of centimeter lesions in the context of Graves and dominant nodules in multinodular goiters (9).

Likewise, we have adopted the 2015 ATA guidelines for using the IONM. If the role of the device in adult thyroid surgery is well defined, scientific evidence is lacking in pediatric surgical practice: recent studies rarely shown level of statistical significance due to the small sample size (25–27). However, the use of IONM appears to be supported and justified on an anatomical basis (6–28): the operating field of a very young child (under 10 years of age) differs considerably in an older child and in adults, and the restricted anatomical space necessarily makes isolation and preservation of the recurrent laryngeal nerve more difficult. According to ATA guidelines, the use of IONM should be considered in children who are less than 10 years of age and in pediatric patients who are candidates for total thyroidectomy with lymphadenectomy and central cervical compartment dissection (9). Similarly, other studies also recommend the use of the nerve control device in other more complex surgical cases, such as the treatment of bulky thyroid carcinomas, Graves’ disease and subjects eligible for completion thyroidectomy (29–31).

In our work we adopted the intermittent neuromonitoring only in selected cases and in patients with thyroid diseases at increased risk of recurrent nerve injury: we have used the device in all children aged 11 or less and in cases of thyroid cancer and Graves-Basedow disease.

In literature, data relating to post-operative complications of pediatric thyroid surgery are often discordant and not very comparable due to the difference selection criteria used in case series. The incidence of recurrent nerve injuries seems to vary between 1.5 and 4.5% in the most recent studies (6), up to percentages of even 17% and 28% in the most antiquated studies (32–34). In our work, we have not recorded any recurrent laryngeal nerve injury, neither as transient nor permanent paralysis both in the group of children undergoing surgery with intermittent IONM and in those without it.

This observation suggests that the visual intraoperative identification of the nerve is essential for the correct preservation of recurrent laryngeal nerve and that the control with an electrical device is only supportive. The IONM represents a valid aid for the surgeon to make easier the identification of the nerve and also providing complementary neurophysiological data, such as the identification of accessory nerve branches or the identification of superior laryngeal nerve.

We didn’t have any cases of postoperative bleeding, hematoma, and surgical site infection. The most frequent complication documented among pediatric patients undergoing thyroid surgery in our Center was found to be transient hypoparathyroidism, with an incidence rate of 24%. Several definitions of this condition are given in literature and this makes it difficult to compare studies with each other (12) In most cases, post-surgical hypoparathyroidism occurs almost immediately after surgery (24-48 hours), and it is defined transient if it does not exceed 4-6 weeks after surgery. It becomes chronic and permanent when persists for more than six months and requires long-term supportive care. Studies in literature report extremely variable incidences of transient hypoparathyroidism after thyroid surgery, with values between 0.4 and 26.8% after subtotal thyroidectomy (12–36) and values between 19 and 38% after total thyroidectomy (12–37).

According to recent literature, there are several factors involved in the development of transient postoperative hypoparathyroidism in adult population: among these, the most mentioned are female gender, young age (under 12), underlying thyroid disease (Graves’ disease), type of surgical procedures performed (TT or lobectomy), experience of the surgeon and lastly the surgical Center (high volume or not) (38, 39). Other studies report completely different clinical and laboratory parameters, including time required for surgery, estimated weight of the residual gland, and level of PTH and preoperative serum calcium (40). Recent studies still report how total thyroidectomy and central neck compartment dissection are associated with a higher rate of transient hypoparathyroidism (41, 42). Even if risk factors for transient hypoparathyroidism in adults are known, the role of these remains controversial for pediatric population, where data are limited.

In order to avoid selection bias on patients enrolled in the study, we performed preoperative checks with measurement of serum calcium and PTH on all patients. This allowed us to exclude any unrecognized defects related to calcium metabolism and allowed a better patient management in postoperative period: The preoperative dosage of PTH would be useful as a predictor of postoperative hypocalcemia, optimizing the postoperative course with a targeted integrative therapy (43).

In our study, none of the variables considered reached the estimated level of significance, therefore it cannot be correlated with post-operative outcome obtained. Neither gender nor the age of the patient was found to be risk factors for the development of postoperative transient hypoparathyroidism. Similarly, statistical significance was not reached either for thyroid pathology with a surgical indication (Graves’ disease or thyroid carcinoma) nor type of intervention performed (total or partial thyroidectomy).

The systematic research of parathyroid glands, their identification and the careful preservation of vascular peduncle remain fundamental in the intraoperative phase of any thyroidectomy. Leiker AJ et al. report in their work how even the simple non-traumatic manipulation of the parathyroid gland during a total thyroidectomy can cause a transient altered release of PTH with a consequent temporary decrease in calcium levels (44).

Finally, as already mentioned, several studies show how high-volume surgeons had lower complication rates and a lower incidence of hypoparathyroidism than colleagues with lower case series or lower-volume hospitals (45).

## Conclusion

Since January 2019 our Center has started performing thyroid surgery in children with results comparable to the pediatric surgery Centers. Pediatric thyroid nodules show higher malignancy rates and worse prognosis than adults. In our case series the malignancy rate reaches 40% and 75% of lesions typified as TIR 3B at FNA has malignant diagnosis on definitive histological examination. The clinical presentation and the greater regional invasiveness of pediatric thyroid carcinoma therefore require specific and dedicated guidelines.

In our experience pediatric thyroid surgery is associated with a high rate of transient post-surgical hypoparathyroidism (24%), with subsequent normalization of calcium levels in 83% of cases within 30 days of the surgery. The hypocalcemia was biochemical feedback, without symptoms related. We had not major complication such as laryngeal nerve lesions, bleeding or tracheostomy.

The intraoperative finding of a thinner recurrent laryngeal nerve in younger patients makes nerve isolation more difficult than in adult surgery: we recommend IONM in patients younger than 12 years. Pediatric thyroidectomy is a complex surgery: we suggest multidisciplinary team with high volume endocrine surgeons, pediatric endocrinologists, anesthetists and pathologists dedicated.

## Data availability statement

The raw data supporting the conclusions of this article will be made available by the authors, without undue reservation.

## Ethics statement

Ethical review and approval was not required for the study on human participants in accordance with the local legislation and institutional requirements. Written informed consent to participate in this study was provided by the participants’ legal guardian/next of kin.

## Author contributions

AB collected, analyzed and interpreted patient data and he was the major contributor in writing the manuscript. FQ reviewed the entire manuscript and supervised the final work. All authors contributed to the article and approved the submitted version.
